# How Phosphofructokinase-1 Promotes PI3K and YAP/TAZ in Cancer: Therapeutic Perspectives

**DOI:** 10.3390/cancers14102478

**Published:** 2022-05-18

**Authors:** Luca Simula, Marco Alifano, Philippe Icard

**Affiliations:** 1 Department of Infection, Immunity and Inflammation, Cochin Institute, INSERM U1016, CNRS UMR8104, University of Paris, 75014 Paris, France; luca.simula@inserm.fr; 2 INSERM U1138, Integrative Cancer Immunology, University of Paris, 75006 Paris, France; marco.alifano@aphp.fr; 3 Service de Chirurgie Thoracique, Hôpital Cochin, Hôpitaux Universitaires Paris Centre, APHP, Paris-Descartes University, 75014 Paris, France; 4 UNICAEN, INSERM U1086 Interdisciplinary Research Unit for Cancer Prevention and Treatment, Normandie Université, 14000 Caen, France

**Keywords:** PFK1, F-1,6-BP, PI3K, YAP/TAZ, drug resistance, citrate

## Abstract

**Simple Summary:**

We propose that PFK1 promotes a positive feedback loop with PI3K/AKT and YAP/TAZ signaling pathways in cancer cells. Therefore, targeting PFK1 (or its product F-1,6-BP) could improve the efficacy of PI3K and YAP/TAZ inhibitors currently tested in clinical trials. To this aim, we suggest the use of citrate, which is a physiologic and potent inhibitor of PFK1.

**Abstract:**

PI3K/AKT is one of the most frequently altered signaling pathways in human cancers, supporting the activation of many proteins sustaining cell metabolism, proliferation, and aggressiveness. Another important pathway frequently altered in cancer cells is the one regulating the YAP/TAZ transcriptional coactivators, which promote the expression of genes sustaining aerobic glycolysis (such as WNT, MYC, HIF-1), EMT, and drug resistance. Of note, the PI3K/AKT pathway can also regulate the YAP/TAZ one. Unfortunately, although PI3K and YAP inhibitors are currently tested in highly resistant cancers (both solid and hematologic ones), several resistance mechanisms may arise. Resistance mechanisms to PI3K inhibitors may involve the stimulation of alternative pathways (such as RAS, HER, IGFR/AKT), the inactivation of PTEN (the physiologic inhibitor of PI3K), and the expression of anti-apoptotic Bcl-xL and MCL1 proteins. Therefore, it is important to improve current therapeutic strategies to overcome these limitations. Here, we want to highlight how the glycolytic enzyme PFK1 (and its product F-1,6-BP) promotes the activation of both PI3K/AKT and YAP/TAZ pathways by several direct and indirect mechanisms. In turn, PI3K/AKT and YAP/TAZ can promote PFK1 activity and F-1,6-BP production in a positive feedback loop, thus sustaining the Warburg effect and drug resistance. Thus, we propose that the inhibition of PFK1 (and of its key activator PFK2/PFKFB3) could potentiate the sensitivity to PI3K and YAP inhibitors currently tested. Awaiting the development of non-toxic inhibitors of these enzymes, we propose to test the administration of citrate at a high dosage, because citrate is a physiologic inhibitor of both PFK1 and PFK2/PFKFB3. Consistently, in various cultured cancer cells (including melanoma, sarcoma, hematologic, and epithelial cancer cells), this “citrate strategy” efficiently inhibits the IGFR1/AKT pathway, promotes PTEN activity, reduces Bcl-xL and MCL1 expression, and increases sensitivity to standard chemotherapy. It also inhibits the development of sarcoma, pancreatic, mammary HER^+^ and lung RAS-driven tumors in mice without apparent toxicities.

## 1. Introduction

The Phosphatidylinositol-3-Kinase (PI3K)/ Protein Kinase B (AKT) pathway is one of the most frequently altered pathways in human cancers [1,2]. Two recently identified downstream targets of PI3K/AKT are the transcriptional coactivators Yes-associated protein (YAP) and its homolog WW-domain-containing transcription regulator 1 (WWTR1; also known as TAZ) (thereafter indicated as YAP/TAZ), which promote multiple transcription factors regulating cell development [3,4,5]. In a wide variety of cancers, the deregulation and activation of PI3K/AKT and YAP/TAZ signaling is nowadays considered as a key event supporting mechano-transduction (shear forces, elasticity, and tissue stretching) [3,6], metabolism, cell cycle progression, survival, epithelial-mesenchymal transition (EMT), metastasis, and drug resistance (including immunotherapy) [3,4,5,7,8,9,10,11]. Therefore, there is an increasing interest in the development of potent PI3K and YAP inhibitors, especially to target various highly resistant cancers (both solid tumors and hematologic malignancies) [2,7,12,13]. However, the occurrence of resistance mechanisms to PI3K and YAP inhibitors may reduce their therapeutic effectiveness. Additionally, PI3K inhibitors have principally cytostatic effect (tumor stabilization rather than tumor regression) [7,12], whereas the efficiency and toxicities of YAP inhibitors need further research [4]. For example, resistance to PI3K/AKT and YAP/TAZ inhibitors can be supported by multiple mechanisms involving their direct activation or the inactivation of upstream negative regulators, such as:

(i) gain function mutations, in particular of PI3K or membrane receptors, such as receptor tyrosine kinases (RTKs), promoting PI3K activity [2,14];
(ii) compensatory activation of pathways able to bypass the PI3K inhibition, such as the activation of JAK2/STAT5 [15] and/or insulin dependent pathways (promoted by insulin-like growth factor 1 (IGF1), insulin-like growth factor receptor 1 (IGFR1), and insulin receptor substrate 2 (IRS2)) [7,16];
(iii) upregulation of key pro-survival factors, such as anti-apoptotic Bcl-xL and MCL1 proteins [17];
(iv) activation of key mutated oncogenes (gain of function), particularly EGFR [8], HER/ERK [18], Kirsten Rat Sarcoma homolog (RAS) [8,19], promoting constitutive activation of PI3K/AKT signaling;
(v) loss of function or epigenetic silencing of suppressor genes, such as Phosphatase and TENsin homolog (PTEN) protein, the key physiologic inhibitor of PI3K [2,20,21];
(vi) inactivation of the Hippo pathway that inhibits YAP/TAZ, a deregulation promoting resistance to inhibitors of V-raf murine sarcoma viral oncogene homolog B (BRAF) and mitogen-activated protein kinase (MAPK) in BRAF mutant cancer cells [4,5,22];
(vii) amplification of the WWTR1 (encoding TAZ) and YAP1 genes or mutations and deletions of the FAT1 gene (a tumor suppressor, whose inactivation favors TAZ nuclear translocation), as reported for head and neck squamous cell carcinoma (HNSCC) [23];
(viii) YAP1 gene fusions, as observed in different tumors (for a review see [24]). YAP1 fusion proteins (normally constituted by N-terminal YAP1 and C-terminal part of another protein) can retain a TEAD-dependent YAP activity and can show resistance to inhibition by the Hippo pathway;
(ix) activating point mutations in TAZ gene, which can transform TAZ into an oncogene, as observed in muscle cells [25].

Consequently, to increase the effectiveness of PI3K/AKT and YAP inhibitors, there is an urgent need to target these various resistance mechanisms possibly through a missing link with PI3K/AKT and YAP/TAZ, as recently suggested [3]. To this aim, we here propose to consider the role of phosphofructokinase-1 (PFK1), the key regulatory enzyme of glycolysis. As we will see, PFK1 and its product fructose-1,6-bisphosphate (F-1,6-BP) can activate both PI3K and/or YAP/TAZ by independent mechanisms. In turn, PI3K/AKT and YAP/TAZ can promote PFK1 and/or glycolysis in a feedback loop [26]. Thus, a “Gordian Knot” of interconnections is created between PFK1/F-1,6-BP, PI3K/AKT, and YAP/TAZ pathways that sustains the Warburg effect (i.e., aerobic glycolysis even in the presence of oxygen) [27], and thus proliferation, aggressiveness, and drug resistance [28]. Therefore, targeting PFK1, as well as its key activator fructose-2,6-bisphosphate (F-2,6-BP) produced by phosphofructokinase-2 (PFK2) (also called 6-phosphofructo-2-kinase/fructose-2,6-bisphosphatase-3 (PFKFB3) in cancer cells), could be a novel and efficient strategy to increase the sensitivity to PI3K and YAP inhibitors. Awaiting the development of non-toxic inhibitors of PFK1 and PFKFB3, we will emphasize how in cultured cancer cells of diverse origins, the targeting of PFK1 has been achieved by the administration of high dosages of citrate, a physiologic inhibitor of PFK1. This “citrate strategy” efficiently inhibits the IGFR1/AKT pathway, promotes PTEN activity, reduces Bcl-xL and MCL1 expression, and increases sensitivity to standard chemotherapy. Oral administration of sodium citrate inhibits the development of sarcoma, pancreatic, HER^+^ mammary and RAS-driven lung tumors in mice models without apparent toxicities [29,30,31]. It also neutralizes the acidity of the tumor microenvironment [32], a condition that decreases the efficiency and penetration of many anti-cancer agents [33,34].

## 2. The Activation of PI3K/AKT and YAP/TAZ Pathways

**Figure 1:**
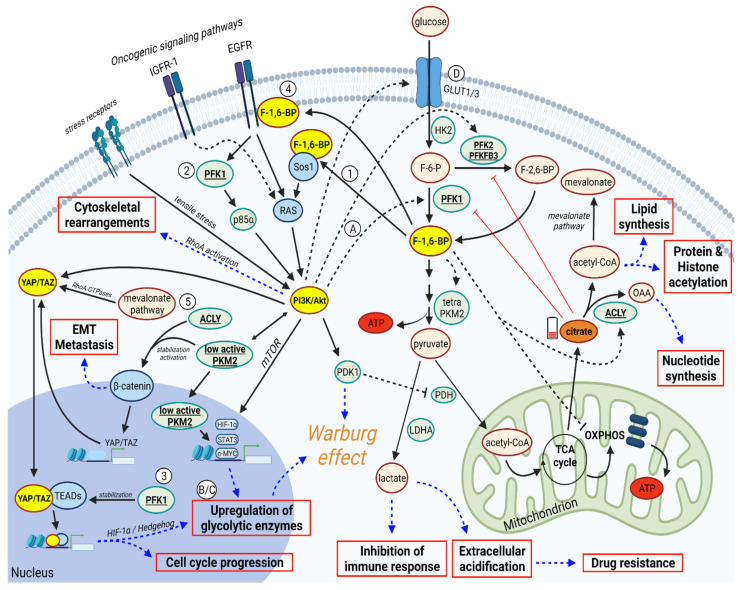
PI3K/AKT, YAP/TAZ, and PFK1/F-1,6-BP create a “Gordian Knot”, supporting cancer progression. In cancer cells, F-1,6-BP promotes glycolysis and activates ACLY, reducing citrate levels (which could inhibit PFK1 and PFK2) and increasing cytosolic acetyl-CoA (promoting lipid synthesis, protein and histone acetylation, and mevalonate pathway) and oxalacetate (OAA, sustaining nucleotide synthesis). Also, F-1,6-BP activates PI3K/AKT through Ras induction. PI3K/AKT can be also activated by tensile stresses from stress receptors. Once activated, PI3K/AKT promote transcription of glycolytic genes (though mTOR and low active PKM2), β-catenin stabilization (through PKM2, also promoted by ACLY), activation of glycolytic enzymes (favoring glycolysis), and YAP/TAZ activation (also induced by mevalonate pathway and β-catenin). Once translocated in the nucleus, YAP/TAZ mediate the transcription of genes promoting cell cycle progression and glycolysis. In parallel, (*i*) F-1,6-BP and AKT inhibit OXPHOS, thus increasing lactate production, which in turn inhibits the immune response and promotes extracellular acidification and drug resistance, and (*ii*) EMT is promoted by β-catenin and YAP/TAZ. Therapeutic strategies increasing citrate levels in cancer cells can inhibit glycolysis and F-1,6-BP production. Therefore, they may be useful to disrupt the Gordian Knot orchestrated by PI3K/AKT, YAP/TAZ, and PFK1/F-1,6-BP. Numbers (1–5) and uppercase letters (A–D) refer to regulatory mechanisms as described in Section 3 (numbers) and Section 4 (letters). Figure created with Biorender.

PI3K is a key transducer of extracellular stimuli, such as growth factors and mechanical stress [3]. After activation by RTKs, G-protein-coupled receptors (GPCR) sensible to various factors (including hormones, metabolites such as purine, adenosine), or by proto-oncogenes RAS-GTPases proteins (Figure 1), PI3Ks are activated and catalyze the phosphorylation of phosphatidylinositol-4,5-bisphosphate-2 (PIP2) into phosphatidylinositol-4,5-bisphosphate-3 (PIP3). Then, PI3P recruits and activates AKT, a kinase that regulates various downstream pathways sustaining cancer cells metabolism, especially the mammalian target of rapamycin (mTOR), sustaining protein translation [35], cell cycle progression, survival, and EMT (for a review, see [1,2]). Importantly, AKT can be considered as “the Warburg kinase” [36] because it activates the mitochondrial pyruvate dehydrogenase kinase-1 (PDK1), which inhibits the pyruvate dehydrogenase (PDH) enzyme [37]. Consequently, pyruvate does not feed the Krebs cycle and is oriented towards lactate production by lactate dehydrogenase A (LDHA). Importantly, lactate secreted in a tumor microenvironment (TME) inhibits the immune response [38] and favors the establishment of an acidic pH that counteracts the efficacy of many anti-cancer agents (including immunotherapies and mTOR inhibitors) [33,34,38,39]. Among the three class of PI3Ks, class IA and IB isoforms have been the most extensively studied. They are heterodimers formed of a catalytic subunit (a p110α and p110β, respectively), associated with a regulatory subunit (most often p85α isoform) [7]. Schematically, class IA isoforms mediate signaling downstream of RTKs, and class IB isoforms mediate signaling downstream of GPCRs [7,12]. Importantly, the PI3K/AKT signaling pathway can be activated even in the absence of extracellular stimuli. This activation can be driven by genetic alterations (gain of function mutations or amplification), in particular of the PI3K p110α catalytic subunit (also named PIK3CA [14]) or of membrane receptors promoting PI3K activity, such as RTKs, including EGFR [2,8,14]. Also, the frequent loss of function or epigenetic silencing of the suppressor PTEN promotes PI3K because PTEN physiologically counteracts the activity of the catalytic subunit of PI3K by transforming PIP3 into PIP2 [2,20]. Similarly, the frequent upregulation of the proto-oncogene KRAS protein promotes the activation of PI3K/AKT signaling [8]. The KRAS proto-oncogene is a small G-protein with guanosine triphosphatase (GTPase) activity, which is involved in the transmission of the signal of most RTKs, including EGFR [8,40]. This small GTPase catalyzes the hydrolysis of guanosine triphosphate (GTP) into guanosine biphosphate (GDP). This reaction ensures the shutdown of the signaling because the GDP-bound isoform of KRAS is inactive [41]. Importantly (as we see later), the conversion from an inactive GDP state into an active GTP state needs KRAS to bind to Son of sevenless homolog 1 (SOS1), a protein activated by PFK1 [42]. Once RAS is activated, its downstream targets are engaged in a cascade, such as the PI3K/AKT and the RAS/RAF/MEK/ERK (MAPK) pathways. Both of them promote cancer cell development and resistance [40], partly by inducing YAP/TAZ activity by several independent mechanisms (see below).

YAP and TAZ are transcriptional coactivators encoded by paralogous genes that regulate transcription by binding to the members of the transcriptional enhancer factor (TEA)-domain (TEAD) family. Then, YAP/TAZ-TEAD complexes promote expression of various genes sustaining cell proliferation, migration, and aggressiveness [4,5]. YAP/TAZ are activated downstream of oncogenic pathways such as PI3K/AKT and WNT, and are also promoted by inactivation of suppressors, such as their key regulator known as the Hippo pathway [5,10,43,44]. Indeed, in normal tissues, YAP/TAZ signaling is downregulated by the core components of the Hippo pathway formed by a cascade of kinases (MST1/2 and LATS1/2) that coordinates organ growth and homeostasis together with nutrition and metabolism [45]. Mechanistically, LATS kinases are activated by MST1/2 (*drosophila* Hippo) or MAP4K4 through phosphorylation [45]. In case of an active control by the Hippo pathway, YAP/TAZ are inactivated by LATS. Phosphorylated YAP/TAZ proteins are retained in the cytoplasm, and then degraded by proteasomal ligase [43,45,46]. In contrast, lack of inhibition by the Hippo pathway favors the translocation of YAP/TAZ into the nucleus, and their further binding to TEAD proteins to form YAP/TAZ-TEAD complexes. Of note, this mechanism has been reported to promote the loss of contact inhibition of cell growth in cancer cells in a PI3K-dependent way [47]. Indeed, activation of PI3K and phosphoinositide-dependent kinase-1 downstream of EGF receptor can induce the dissociation of the Hippo core complex and the consequent inactivation of LATS, dephosphorylation of YAP, and YAP nuclear accumulation and transcriptional activation [47]. In addition to PI3K, also AKT can increase the activity of YAP proteins through phosphorylation of MST1/2, which inhibits their dimerization and activation [44]. Once in the nucleus, YAP/TAZ-TEAD complexes cooperate with other transcription factors (including AP-1, E2F, MYC) to regulate gene transcription [48]. In addition, YAP/TAZ can bind to other transcriptional factors, including β-catenin, to upregulate the expression of genes favoring EMT (such as *Slug*, *Snail*, *RhoA*) and cell survival (such as *Bcl-xL, survivin*). Also, YAP downregulates PTEN by inducing miR-29, which inhibits PTEN translation [49]. Of note, in diabetic mice with nephropathy, YAP protein can promote its activation in a feedback loop by inhibiting the Hippo pathway, thus promoting its nuclear accumulation and transcriptional effect resulting in an increased proliferation of glomerular mesangial cells [50].

YAP/TAZ activation can promote cancer development in several ways (for a review see [51]). First, YAP/TAZ can suppress apoptotic pathway of cell death by upregulating Bcl-2 family members [52]. Second, sustained activation of YAP/TAZ can promote aberrant cell cycle proliferation, partly through the activation of proto-oncogenes, such as cMYC [53], or through loss of contact inhibition of cell growth [45,46,47]. Third, YAP/TAZ activation has been associated with the reprogramming of tumor cells into tumor cancer stem cells (CSCs) [54]. A positive correlation between YAP/TAZ levels and cancer malignancy (including lower response to therapy), or a higher YAP/TAZ expression level compared to normal tissue, has been observed in several human cancer or murine models, including colorectal, liver, lung, pancreatic, gastric, and breast cancer (see [51] for a review). As stated before, genetic alterations have been observed in YAP/TAZ genes in cancer cells, such as gene amplification [23] and gene fusions [24]. Of note, YAP/TAZ may also upregulate PD-L1 in human breast cancer, mesothelioma, melanoma, and non-small cell lung cancer (NSCLC) cells, thus favoring cancer immune evasion [55]. Additional effects of YAP/TAZ on tumoral microenvironment favoring tumor growth or immune escape have also been reported (see [51] for a review).

To summarize, PI3K/AKT and YAP/TAZ pathways are closely interconnected, and their deregulation can drive cell cycle progression, anchorage-independent cell growth, altered metabolism, and cancer cell survival.

## 3. PFK1 and F-1,6-BP Activates PI3K/AKT and YAP/TAZ Signaling Pathways

In this paragraph, we will examine several mechanisms by which PFK1 and F-1,6-BP can activate or modulate the PI3K/AKT and YAP/TAZ signaling pathways (Figure 1):

(1) F-1,6-BP promotes the activation of PI3K/AKT signaling by binding to SOS1, the key guanine nucleotide exchange factor that promotes strong activation of RAS protein [42]. Subsequently, PI3K/AKT is activated downstream of RAS [40]. Importantly, the binding F-1,6-BP/SOS1 is an evolutionary conserved mechanism that links glycolysis to RAS activation in yeast, as well as in mammalian and cancer cells [42].

(2) Platelet isoform of PFK1 (PFKP) mediates PI3K activation downstream of EGF receptor [56]. Mechanistically, EGFR activation resulted in acetylation (on K395), plasma membrane translocation, and phosphorylation (on Y64) of PFKP. Phosphorylated PFKP binds to the N-terminal SH2 domain of p85α, which in turn results in PI3K activation [56]. Of note, PFKP isoform is frequently highly expressed in lung cancer tissues and cell lines, and it is associated with tumor size and patient prognosis [57].

(3) As shown in several human breast cancer cell lines, PFK1 can bind the transcriptional cofactors TEADs, a binding promoting the functional and biochemical cooperation of TEADs with YAP/TAZ [58] and resulting in gene transcription sustaining proliferation and aggressiveness.

(4) In addition to promoting YAP/TAZ through activation of SOS1/RAS/PI3K/AKT axis, F-1,6-BP can also promote YAP through PI3K/AKT independent pathways, by promoting the β-catenin/WNT pathway, for example. Indeed, F-1,6-BP can bind to the epidermal growth factor receptor (EGFR), as shown in triple negative breast cancer cells, thus enhancing EGFR activity [42,59]. The EGFR/ERK pathway induces the translocation of the low active dimeric pyruvate kinase M2 (PKM2) isoform into the nucleus [60], with subsequent β-catenin transactivation and further stimulation of the WNT signaling pathway [61]. In turn, the WNT/ β-catenin pathway promotes YAP/TAZ transcription [61,62]. Remarkably, the WNT/β-catenin pathway also promotes the activation of *c-MYC*, *cyclin D1,* and, through hypoxia-inducible factor-1alpha (*HIF-1α)* and signal transducer and activator of transcription-3 (*STAT3*), the transcription of many glycolytic enzyme genes (*PFK1*, *PFKFB3, PKM2, LDHA*) [61,63,64]. Thus, aerobic glycolysis is enhanced in a hypoxia-independent manner. Of note, alternative WNT signaling (i.e., not involving β-catenin) can also induce YAP/TAZ activation [65].

(5) F-1,6-BP and AKT can cooperate to induce YAP/TAZ activation by promoting the activity of ATP citrate lyase (ACLY) [66,67]. This enzyme transforms cytosolic citrate into oxaloacetate (sustaining nucleotide synthesis) and acetyl-CoA (sustaining histone acetylation and lipid synthesis). Although no formal studies show that ACLY can induce YAP/TAZ activation, we would like to propose that ACLY may promote YAP/TAZ by two indirect mechanisms (but further studies are required to show whether they really occur in cancer cells). First, ACLY can promote the WNT/β-catenin pathway by affecting the stability of β-catenin, and this is known to promote YAP/TAZ transcription [68]. Second, ACLY could sustain YAP/TAZ by providing the acetyl-CoA required for the mevalonate pathway (MVP), which not only controls cholesterol synthesis but can also promote YAP/TAZ activation [69]. Mechanistically, the mevalonate cascade produces the geranylgeranyl-pyrophosphate that is required for activation of Rho GTPases that, in turn, activates YAP/TAZ by inhibiting their phosphorylation, thus promoting their nuclear accumulation, as shown in breast cancer cells [69]. Of note, the transcription of ACLY and MVP genes is induced by the nuclear translocation and activation of the sterol regulatory element-binding protein-1 (SREBP1) (as shown in hepatocarcinoma) [70]. This transcription is promoted by AKT that upregulates SREBP1 at both the transcriptional and post-translational levels (as shown in lung adenocarcinoma) [71]. YAP/TAZ can reinforce the lipogenic effects of PI3K/AKT by interacting with SREBP-1c and SREBP-2 on the promoters of the fatty acid synthase (FAS) and hydroxyl methyl glutaryl coenzyme A reductase (HMGCR), the regulatory enzyme of the MVP which is also inhibited by statins [71,72,73]. 

To summarize, PFK1 and F-1,6-BP can activate PI3K/AKT and YAP/TAZ signaling pathways through different mechanisms.

## 4. In a Feedback Loop, PI3K/AKT and YAP/TAZ can Promote PFK1/F-1,6-BP

Once activated, both PI3K/AKT and YAP/TAZ pathways can activate PFK1 and favor the accumulation of F-1,6-BP by several mechanisms (Figure 1):

(A) PI3K activates AKT, which in turn promotes the activity of PFK1 [26] (as shown in glioblastoma), and also of PFKFB3 (as showed in HeLa cells). The latter is the enzyme producing F-2,6-BP, the key activator of PFK1.

(B) The transcription factor HIF-1α [74] is a key driver of aerobic glycolysis, promoting the expression of many glycolytic genes, including PFK1 [64]. Of note, both PI3K/AKT and YAP can activate HIF-1α (and thus PFK1 transcription) through increasing transcription [75] or protein stabilization [74], respectively.

(C) YAP/TAZ can promote PFK1 activity via a cross-talk with a Hedgehog signaling pathway. This cross-talk induces the transcription of PFKFB3 (producing F-2,6-BP) and hexokinase 2 (HK2), thus increasing the carbon flux towards PFK1 [76].

(D) Both PI3K/AKT and YAP/TAZ favor the glucose uptake by increasing the expression of glucose transporters GLUT1 and/or GLUT3 [77,78,79,80], thus increasing further the carbon flux towards PFK1 (and so F-1,6-BP production).

Overall, by taking into consideration all the cross-regulations so far described, a positive feedback loop can be established between PFK1/F-1,6-B, PI3K/AKT, and YAP/TAZ.

## 5. Discussion

As we have seen, PI3K/AKT and YAP/TAZ signaling, which are frequently activated in resistant cancers, are deeply interconnected. Efficiently targeting these pathways by specific inhibitors alone or in combination is a great challenge, frequently due to different resistance mechanisms. Furthermore, YAP/TAZ can be activated even downstream of a RAS/MAPK or EGFR blockade, thus allowing the inhibitors of these pathways to be bypassed. In this setting, as we have seen, PI3K/AKT and YAP/TAZ can also be activated by PFK1/F-1,6-BP, either through PI3K-dependent or PI3K-independent mechanisms. Thus, targeting the PFK1/F-1,6-BP axis could be fundamental to increase the effectiveness of PI3K/AKT and YAP/TAZ inhibitors, which currently appear modest [7,12]. Of note, a high expression of PFK1 isoform PFKP is related to tumor size, histological grade, lymph node metastasis, and poor patient survival in NSCLC [57,81], which represents the most frequent cause of cancer death in 2020 [82]). Also, a high expression of PFKFB3 is a prognostic factor of poor prognosis in human HER2^+^ breast cancer and it correlates with TNM in lung adenocarcinoma [83].

As we have seen, many studies argue for the presence of a positive feedback loop between PFK1/F-1,6-BP, PI3K/AKT, and YAP/TAZ. This interconnection can conspire to support cancer cell development and resistance to therapeutics agents, particularly to PI3K and YAP inhibitors. In this loop, PFK1/F-1,6-BP appears as an essential link to couple the enhancement of glycolysis with the activation of PI3K/AKT and YAP/TAZ signaling. All these activations join forces to promote the Warburg effect, proliferation, EMT, and drug resistance. Of note, the interaction of RAS with p110α should be required for RAS-driven tumor formation, as shown in experimental studies in mice [84]. In this context, decreasing the production of F-1,6-BP could attenuate SOS1/RAS binding, and thus slowdown the development of cancers driven by RAS proto-oncogene or RAS mutations (this later form being highly drug resistant) [85]. In addition, the acidification of the extracellular environment promoted by the Warburg effect [28] contributes to the exhaustion of the immune response [38] and to drug resistance [33,34,38]. This occurs because the vast majority of drugs (including conventional chemotherapeutics and more recent biological agents) are weak bases that are neutralized in acidic environments [39]. Thus, targeting the Warburg effect could increase drug sensitivity and could be applied to PI3K and YAP/TAZ inhibitors as well.

## 6. Therapeutic Implications

We suggest that targeting PFK1 activity and F-1,6-BP production could help disrupt the here identified Gordian Knot between PFK1, PI3K/AKT, and YAP/TAZ. Of note, this strategy could enhance the efficacy of PI3K and YAP/TAZ inhibitors currently tested in several aggressive solid cancers and hematologic malignancies (listed in [12]). Particularly, this could be important in those cases in which resistance mechanisms to PI3K and/or YAP/TAZ inhibitors arise in cancer cells. In addition, although PFK1 likely acts as a priming factor in this interconnection between metabolism and PI3K/AKT and YAP/TAZ, the inhibition of the PFK1 activator PFK2/PFKFB3 could also be an efficient strategy.

Awaiting the development of specific and non-toxic inhibitors of PFK1 and PFKFB3 [86,87], we propose to test strategies increasing the citrate level in cancer cells, since citrate is a well-known physiologic and potent inhibitor of PFK1 and PFK2 (Figure 1) [88,89]. Citrate is an intermediate metabolite of the TCA cycle. It is produced by citrate synthase (CS) from acetyl-CoA and oxalacetate, and it is subsequently transformed into isocitrate by the enzyme aconitase. Alternatively, citrate can be exported into the cytosol through the mitochondrial citrate carrier (SLC25A1/CIC) [90]. In the cytosol, citrate can be consumed by the ATP Citrate Lyase (ACLY) enzyme to generate acetyl-CoA (which sustains protein and DNA acetylation, as well as lipid synthesis) and oxalacetate (which can sustain gluconeogenesis and nucleotide synthesis). In addition to its involvement in metabolic reactions, citrate can also exert some regulatory functions. Indeed, as previously said, citrate can suppress glycolysis and favor gluconeogenesis, since it is a potent inhibitor of glycolytic enzymes PFK1 and PFK2 [88,89] and enhances the activity of the gluconeogenesis enzyme fructose-1,6-biphosphatase (FBPase, an enzyme counteracting PFK1 activity) [91]. In hepatocytes, a citrate-mediated inhibition of glycolysis can sustain the gluconeogenesis induced by glucagon [30]. We previously proposed that in proliferative cancer cells, a low citrate level should be established [30]. Indeed, in cells relying on aerobic glycolysis (Warburg effect), PI3K/AKT and the glycolytic intermediate F-1,6-BP cooperate to inhibit mitochondrial OXPHOS [37,92], and thus the production of citrate, which is also rapidly consumed by ACLY [30]. This would prevent the potential inhibition of PFK1 and PFK2 by citrate, sustaining aerobic glycolysis further. Of note, according to in vitro studies, dichloroacetate restores sensitivity to Paclitaxel in resistant lung adenocarcinoma cells by inducing citric acid accumulation [93].

By taking into account these considerations, we propose that administration of citrate at high doses could reinforce the efficacy of PI3K and YAP/TAZ inhibitors currently used against cancer cells since (by inhibiting PFK1) it can contribute to disrupt the positive feedback loop between these two signaling pathways and PFK1/F-1,6-BP. Remarkably, as shown in cancer cell lines of various origins (hematologic, sarcoma, melanoma, and epithelial cancers) (for recent review see [30]), the direct administration of sodium citrate at a high concentration (>10 mM) shows various anti-cancer effects. Indeed, citrate at a high concentration efficiently inhibits PFK1 [94], inactivates PI3K/AKT/mTOR [95] and IGFR1/RAS/AKT pathways [29], reduces expression of anti-apoptotic factors (Bcl-xL, MCL1 and survivin) [96,97,98], reduces β-catenin levels [99], attenuates HIF-1α expression [100], induces PTEN expression [29,101], and increases the sensitivity to cisplatin treatment [29,31,96]. Coherently, in A549 cells, ACLY knockdown (increasing citrate level) inhibits PI3K/AKT and reverses cancer stemness and EMT, especially by reducing Snail expression [66,102]. ACLY knockdown also attenuated cisplatin resistance by inhibiting the PI3K-AKT pathway in ovarian cancer cells [103] and in hepatocarcinoma cells. Additionally, it remarkably suppresses stemness properties, migration, and invasion [68]. These results are also corroborated in in vivo studies by oral administration of sodium citrate at high dosages (around 500 mg/kg). Indeed, in mice models, including osteosarcoma and fibrosarcoma [31], prostate cancer [95], RAS-driven lung tumor, breast HER2^+^ cancer, and also pancreatic cancer xenografts [29], this “citrate strategy” administered orally regressed tumor growth in all cases without remarkable toxicity. Furthermore, it enhanced cisplatin anti-tumor effects [31], reversed EMT, increased intra-tumoral lymphocytes infiltration [29], and also neutralized the TME acidity, enhancing the therapeutic effect of 5-fluoro-uracil derivative in a pancreatic cancer xenograft mice model [32]. Lastly, it should be mentioned that citrate has a very low toxicity because it is an endogenous metabolite with a rapid metabolism and a short half-life. Although excessive administration of citrate could lead to muscle spasms, convulsions, hemorrhage, and hypocalcemia, these adverse effects can be treated and prevented by administration of calcium chloride. In addition, it should be remarked that the active dose showing anti-cancer effects in humans should be much lower than the one causing these adverse effects [30]. However, clinical trials should determine the mode and duration of citrate administration to optimize its efficacy while avoiding any side effects on patients.

## 7. Conclusions

In conclusion, we propose to target PFK1/F-1,6-BP to disrupt the “Gordian Knot” linking PFK1/F-1,6-BP, PI3K/AKT and YAP/TAZ, as well as to increase the sensitivity to current PI3K/AKT and YAP/TAZ inhibitors. To this aim, we encourage to test the “citrate strategy” above described in clinical trials given its promising results in preclinical models obtained without remarkable toxicities.
